# Identification and characterization of mutations responsible for the β-lactam resistance in oxacillin-susceptible *mecA*-positive *Staphylococcus aureus*

**DOI:** 10.1038/s41598-020-73796-5

**Published:** 2020-10-09

**Authors:** Tanit Boonsiri, Shinya Watanabe, Xin-Ee Tan, Kanate Thitiananpakorn, Ryu Narimatsu, Kosuke Sasaki, Remi Takenouchi, Yusuke Sato’o, Yoshifumi Aiba, Kotaro Kiga, Teppei Sasahara, Yusuke Taki, Feng-Yu Li, Yuancheng Zhang, Aa Haeruman Azam, Tomofumi Kawaguchi, Longzhu Cui

**Affiliations:** 1 Division of Bacteriology, Department of Infection and Immunity, Jichi Medical University, 3311-1, Yakushiji, Shimotsuke-shi, Tochigi 329-0498 Japan; 2 School of Medicine, Jichi Medical University, 3311-1, Yakushiji, Shimotsuke-shi, Tochigi 329-0498 Japan

**Keywords:** Genetics, Microbiology

## Abstract

*Staphylococcus aureus *strains that are susceptible to the β-lactam antibiotic oxacillin despite carrying *mecA *(OS-MRSA) cause serious clinical problems globally because of their ability to easily acquire β-lactam resistance. Understanding the genetic mechanism(s) of acquisition of the resistance is therefore crucial for infection control management. For this purpose, a whole-genome sequencing-based analysis was performed using 43 clinical OS-MRSA strains and 100 mutants with reduced susceptibility to oxacillin (MICs 1.0–256 µg/mL) generated from 26 representative OS-MRSA strains. Genome comparison between the mutants and their respective parent strains identified a total of 141 mutations in 46 genes and 8 intergenic regions. Among them, the mutations are frequently found in genes related to RNA polymerase (*rpoBC*), purine biosynthesis (*guaA, prs, hprT*), (p)ppGpp synthesis (*rel*_*Sau*_), glycolysis (*pykA, fbaA, fruB*), protein quality control (*clpXP, ftsH*), and tRNA synthase (*lysS, gltX*), whereas no mutations existed in *mec *and *bla *operons. Whole-genome transcriptional profile of the resistant mutants demonstrated that expression of genes associated with purine biosynthesis, protein quality control, and tRNA synthesis were significantly inhibited similar to the massive transcription downregulation seen in *S. aureus *during the stringent response, while the levels of *mecA *expression and PBP2a production were varied. We conclude that a combination effect of *mecA* upregulation and stringent-like response may play an important role in acquisition of β-lactam resistance in OS-MRSA.

## Introduction

*Staphylococcus aureus* is an important bacterial pathogen that can cause life-threatening infections in both humans and animals^1,2^. A known feature of *S. aureus* is its evolutionary potential to develop antibiotic resistance under selection pressure via antibiotic treatment. Methicillin-resistant *S. aureus *(MRSA) is resistant to the entire class of β-lactam antibiotics, including penicillin, methicillin, and cefazolin^3^. It was first recognized as a problematic pathogen in hospital settings, but it has subsequently emerged in community settings and livestock^3–5^. MRSA infections remain a major concern in the clinical setting because they are more difficult to treat than infections caused by other β-lactam-susceptible strains of *S. aureus*. The β-lactam resistance in MRSA is primarily mediated by a non-native *mecA* gene encoding modified penicillin-binding protein 2a (PBP2a), which has an extremely low affinity for β-lactams. The expression of PBP2a is dependent on the presence of functional MecI/MecR1/MecR2 and BlaI/BlaR1 regulators in the *mec* and *bla* operons, respectively^6,7^. However, the level of β-lactam resistance does not always correlate with that of PBP2a expression^6–8^.

Recently, oxacillin-susceptible *mecA*-positive *S. aureus* (OS-MRSA) strains have been increasingly reported worldwide in clinical isolates as well as in animals and food^9–15^. In clinical microbiology laboratories, an oxacillin minimum inhibitory concentration (MIC) ≥ 4 µg/mL or cefoxitin MIC ≥ 8 µg/mL is routinely used as a breakpoint for diagnosing MRSA, whereas the presence of *mecA* has been used as a genetic marker for identification of MRSA^16^. Owing to its susceptibility to oxacillin, OS-MRSA might be misidentified as methicillin-susceptible *S. aureus *(MSSA) in routine clinical laboratories in which *mecA* detection is unavailable^17^. In addition, despite being susceptible to β-lactam antibiotics, OS-MRSA is prone to develop β-lactam resistance following antibiotic therapy due to its carriage of *mecA*^10–12,14,15^, ultimately leading to β-lactam treatment failure^18–20^. Recent findings in study of mechanism of the β-lactam resistance in OS-MRSA include either (1) restoration of frameshift mutation in *mecA* or (2) mutations in other genes that are not directly relevant to function of *mecA*^21–23^. In the present study, a collection of 43 clinical OS-MRSA strains and 100 mutants with reduced susceptibility to β-lactam resistance selected by exposing the OS-MRSA to oxacillin were analyzed to identify genome mutations responsible for β-lactam resistance.

## Results

### Characterization of clinical OS-MRSA isolates

A total of 43 OS-MRSA isolates recovered from various clinical specimens collected from Japan and Taiwan were included in this study (Table S1). The characteristics of OS-MRSA were re-confirmed via determination of their oxacillin susceptibility and the presence of *mecA* (Table 1). Our results revealed that all strains maintained the typical characteristics of OS-MRSA, including *mecA* positivity and susceptibility to oxacillin, but they were susceptible to oxacillin with MICs ranging from 0.125 to 2 µg/mL. According to CLSI, cefoxitin can also be used to detect MRSA. Thus, cefoxitin susceptibility testing was conducted to investigate whether there was discrepancy between oxacillin and cefoxitin susceptibility among the OS-MRSA isolates. The cefoxitin MICs for all OS-MRSA isolates ranged 1.5–12 µg/mL, with 24 of the 43 OS-MRSA isolates (56%) exhibiting susceptibility to cefoxitin.

**Table 1:** Characteristics of the 43 clinical OS-MRSA isolates.

Clade^a^	Strain	MIC (μg/mL)		MLST								SCC*mec* typing			
		Oxacillin	Cefoxitin	*arcC*	*aroE*	*glpF*	*gmk*	*pta*	*tpi*	*yqiL*	ST^b^	SCC*mec* type	*mecR1*	*mecI*	*mecA*^d^
1	JMUB1297	0.25	1.5	3	3	1	1	4	4	3	8	IVa	+	ND^c^	+
	JMUB1282	0.125	2	3	3	1	1	4	4	3	8	IVa	+	ND	+
	JMUB1315	0.25	3	3	3	1	42	4	4	3	1516	IVc	+	ND	+
2	JMUB492	2	12	1	1	1	1	1	1	1	1	IVa	+	ND	+
3	JMUB217	0.75	4	1	1	1	1	22	1	1	772	V	+	ND	+
4	JMUB1293	0.125	1.5	1	4	1	4	12	1	10	5	II	+	+	+
5	JMUB1308	0.5	4	1	26	28	18	18	33	50	89	IVa	+	ND	+
	JMUB1311	0.19	4	1	26	28	18	18	33	50	89	IVa	+	ND	+
	JMUB1291	1.5	6	1	26	28	18	18	33	50	89	IVa	+	ND	+
	JMUB1305	0.5	4	1	26	28	18	18	54	50	91	IVa	+	ND	+
	JMUB1285	0.5	6	1	26	28	18	18	33	50	89	V	+	ND	+
	JMUB1289	0.5	6	1	26	28	18	18	33	50	89	V	+	ND	+
	JMUB1284	0.5	4	1	26	28	18	18	33	50	89	V	+	ND	+
	JMUB1283	0.38	4	1	26	28	18	18	33	50	89	V	+	ND	+
	JMUB1304	0.75	4	1	26	28	18	18	33	50	89	V	+	ND	+
	JMUB1301	1	6	1	26	28	18	18	33	50	89	V	+	ND	+
6	JMUB1973	0.75	6	19	23	15	2	19	20	15	59	V	+	ND	+
	JMUB1981	1	6	19	23	15	2	19	20	15	59	V	+	ND	+
	JMUB1980	0.5	4	19	23	15	2	19	20	15	59	V	+	ND	+
	JMUB1976	1.5	6	19	23	15	2	19	20	15	59	V	+	ND	+
	JMUB1978	0.75	6	19	23	15	2	19	20	15	59	V	+	ND	+
	JMUB1972	1	6	19	23	15	2	19	20	15	59	V	+	ND	+
	JMUB1974	0.25	1.5	19	23	15	2	19	20	15	59	V	+	ND	+
	JMUB1977	0.75	6	19	23	15	48	19	20	15	338	V	+	ND	+
	JMUB1979	0.5	8	19	23	15	2	19	20	15	59	IVa	+	ND	+
7	JMUB1298	0.75	8	6	5	6	2	7	14	5	121	V	+	ND	+
	JMUB1303	0.25	4	6	5	6	2	7	14	5	121	V	+	ND	+
	JMUB1281	1	6	6	5	6	2	7	14	5	121	V	+	ND	+
	JMUB1286	1	6	6	5	6	2	7	14	5	121	V	+	ND	+
	JMUB1300	0.19	1.5	6	5	6	2	7	14	5	121	V	+	ND	+
	JMUB1314	0.75	6	6	5	6	2	7	14	5	121	V	+	ND	+
	JMUB1312	1	4	6	5	6	2	7	14	5	121	V	+	ND	+
	JMUB1313	0.75	2	6	5	6	2	7	14	5	121	V	+	ND	+
	JMUB1288	0.5	2	6	5	6	2	7	14	5	121	V	+	ND	+
	JMUB1280	0.5	4	6	5	6	2	7	14	5	121	V	+	ND	+
	JMUB1295	0.19	3	6	5	6	2	7	14	5	121	V	+	ND	+
	JMUB1299	0.25	4	6	5	6	2	7	14	5	121	V	+	ND	+
	JMUB1316	0.75	6	6	5	6	2	7	14	5	121	V	+	ND	+
	JMUB1294	0.38	6	6	5	6	2	7	14	5	121	V	+	ND	+
	JMUB1296	0.5	4	6	5	6	2	7	14	5	121	V	+	ND	+
	JMUB1292	0.75	4	6	5	6	2	7	14	5	121	V	+	ND	+
	JMUB1310	0.5	4	73	5	6	2	7	14	5	6217	V	+	ND	+
	JMUB1302	1	6	73	5	6	2	7	14	5	6217	V	+	ND	+

Clade^a^	Strain	*bla* operon-1				*bla* operon-2									
		*blaR1*	*blaI*	*blaZ*	Location	*blaR1*	*blaI*	*blaZ*	Location						
1	JMUB1297	ND	ND	ND	–	ND	ND	ND	–						
	JMUB1282	+	+	+	Plasmid	ND	ND	ND	–						
	JMUB1315	ND	ND	ND	–	ND	ND	ND	–						
2	JMUB492	+	+	+	Plasmid	ND	ND	ND	–						
3	JMUB217	+	+	+	Chromosome	+	+	+	Chromosome						
4	JMUB1293	ND	ND	ND	–	ND	ND	ND	–						
5	JMUB1308	ND	ND	ND	–	+	+	+	Chromosome						
	JMUB1311	ND	ND	ND	–	+	+	+	Chromosome						
	JMUB1291	ND	ND	ND	–	+	+	+	Chromosome						
	JMUB1305	ND	ND	ND	–	+	+	+	Chromosome						
	JMUB1285	ND	ND	ND	–	+	+	+	Chromosome						
	JMUB1289	ND	ND	ND	–	+	+	+	Chromosome						
	JMUB1284	ND	ND	ND	–	+	+	+	Chromosome						
	JMUB1283	ND	ND	ND	–	+	+	+	Chromosome						
	JMUB1304	ND	ND	ND	–	+	+	+	Chromosome						
	JMUB1301	ND	ND	ND	–	+	+	ND	Chromosome						
6	JMUB1973	+	+	+	Plasmid	ND	ND	ND	–						
	JMUB1981	+	+	+	Plasmid	ND	ND	ND	–						
	JMUB1980	+	+	+	Plasmid	ND	ND	ND	–						
	JMUB1976	+	+	+	Plasmid	ND	ND	ND	–						
	JMUB1978	+	+	+	Plasmid	ND	ND	ND	–						
	JMUB1972	+	+	+	Plasmid	ND	ND	ND	–						
	JMUB1974	+	+	+	Plasmid	ND	ND	ND	–						
	JMUB1977	+	+	+	Plasmid	ND	ND	ND	–						
	JMUB1979	+	+	+	Plasmid	ND	ND	ND	–						
7	JMUB1298	ND	ND	ND	–	ND	ND	ND	–						
	JMUB1303	ND	ND	ND	–	+	+	+	Chromosome						
	JMUB1281	ND	ND	ND	–	+	+	+	Chromosome						
	JMUB1286	ND	ND	ND	–	+	+	+	Chromosome						
	JMUB1300	ND	ND	ND	–	+	+	+	Chromosome						
	JMUB1314	ND	ND	ND	–	+	+	+	Chromosome						
	JMUB1312	ND	ND	ND	–	+	+	+	Chromosome						
	JMUB1313	ND	ND	ND	–	+	+	ND	Chromosome						
	JMUB1288	ND	ND	ND	–	+	+	+	Chromosome						
	JMUB1280	ND	ND	ND	–	+	+	+	Chromosome						
	JMUB1295	ND	ND	ND	–	+	+	+	Chromosome						
	JMUB1299	ND	ND	ND	–	+	+	+	Chromosome						
	JMUB1316	ND	ND	ND	–	ND	ND	ND	–						
	JMUB1294	ND	ND	ND	–	ND	ND	ND	–						
	JMUB1296	ND	ND	ND	–	ND	ND	ND	–						
	JMUB1292	ND	ND	ND	–	+	+	+	Chromosome						
	JMUB1310	ND	ND	ND	–	+	+	+	Chromosome						
	JMUB1302	ND	ND	ND	–	+	+	+	Chromosome						

^a^Clade classified from a particular branch on the phylogenetic tree.

^b^Sequence type.

^c^Not detected.

^d^Detected by PCR and whole genome sequencing data.

### Genomic analysis of the clinical OS-MRSA isolates

**Figure 1:**
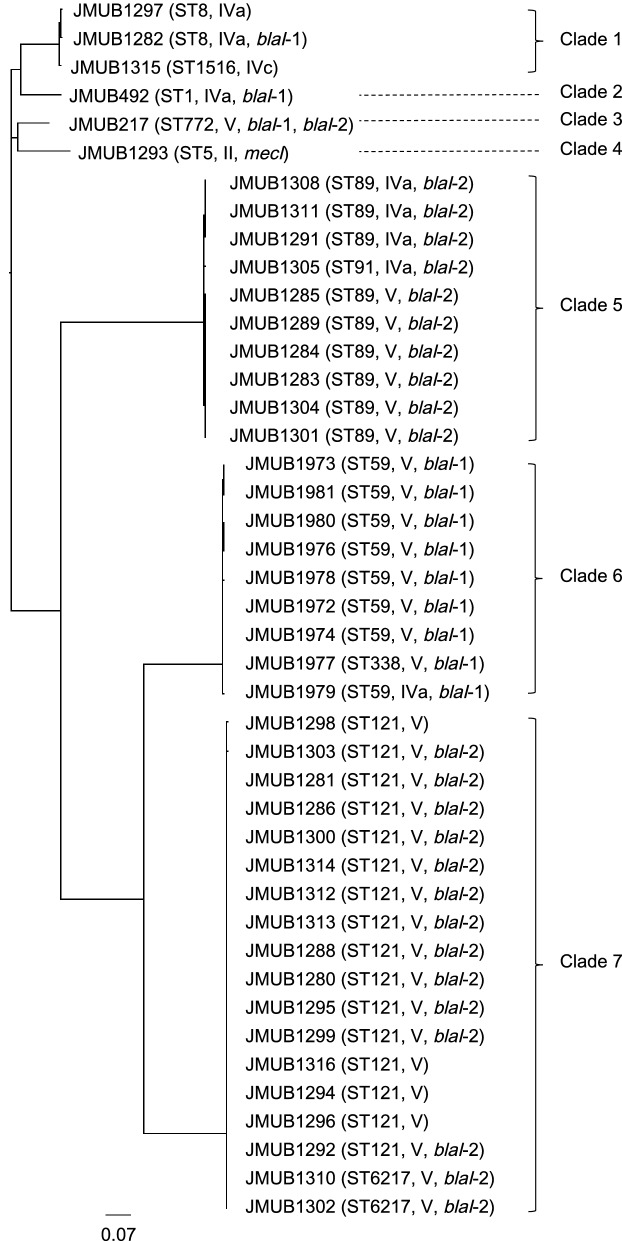
Phylogenetic relationships among clinical isolates of oxacillin-susceptible *mecA*-positive *S. aureus* (OS-MRSA). A maximum parsimony tree of 43 OS-MRSA isolates was generated with the majority of single nucleotide polymorphisms in the core genome using FigTree ver.1.4.3. The sequence type (ST) of MLST, SCC*mec* type, and *blaI* genotypes of each strain were appended to this figure. OS-MRSA isolates were classified into seven main clades (clades 1–7).

To determine the genetic background of the strains used in this study, the whole-genome sequences of the 43 clinical OS-MRSA isolates were determined, and their phylogenetic relationships were analyzed by constructing a phylogenetic tree using kSNP3 (Fig. 1). The phylogenetic tree revealed extensive genomic diversity among the isolates, which could be classified into seven main phylogenetic clades. In addition, these isolates could also be grouped into 11 MLST types (ST1, ST5, ST8, ST59, ST89, ST91, ST121, ST338, ST772, ST1516, and ST6217), and they carried four different SCC*mec* types (II, IVa, IVc, and V). The majority of OS-MRSA isolates were belonged to ST121-SCC*mec* type V (16 strains, 37%), followed by ST59-SCC*mec* type V (seven strains, 16%), ST89-SCC*mec* type V (six strains, 14%), ST89-SCC*mec* type IVa (three strains, 7.0%), ST8-SCC*mec* type IVa (two strains, 4.7%), and ST6217-SCC*mec* type V (two strains, 4.7%). In addition, seven singletons (ST1-SCC*mec* type IVa, ST5-SCC*mec* type II, ST59-SCC*mec* type IVa, ST91-SCC*mec* type IVa, ST338-SCC*mec* type V, ST772-SCC*mec* type V, and ST1516-SCC*mec* type IVc), each of which comprised 2.3% of all strains, were identified. SCC*mec* types V (33 strains, 77%) and IVa (eight strains, 19%) were predominant among the OS-MRSA isolates, whereas only one isolate harbored each of SCC*mec* type II and IVc, respectively. Single nucleotide polymorphisms (SNPs) found in promoter and coding region of *mecA* are listed in Table 2. The type of SNPs located on *mecA* promoter region, consisting of MecI/BlaI-binding site (− 19 to − 50) and ribosome-binding site (− 7 to − 11)^24^, and coding region were closely correlated with SCC*mec* types. For the SNPs in promotor region, SCC*mec* type II strain JMUB1293 carried an C-30A mutation (A replace C of 30th bases upstream of *mecA* CDS), all 8 strains belong to SCC*mec* type IVa had G-7 T mutations, and all strains belong to SCC*mec* type IVc (1 strain) and V strains (34 strains) carried C-33 T mutations. For the SNPs in *mecA* coding region, all OS-MRSA strains had a synonymous mutation of C75A, and all SCC*mec* type V strains carried nonsynonymous mutations of T675A (Ser225Arg), when compared to a prototypic pre-MRSA strain N315, which carries intact *mec* operon (composed of *mecA, mecI,* and *mecR1*) and its *mecA* gene expression is strongly repressed by *mecI*^25^.

**Table 2:** Nucleotide polymorphism on *mecA* promoter and coding region of the 43 clinical OS-MRSA isolates.

Strain	ST^b^	SCC*mec* type	Identity	Nucleotide polymorphism in *mecA* gene at position^a^								
				− 33	− 30	− 20	− 7	+ 75	+ 130	+ 281	+ 675	+ 737
N315	5	II		C	C	C	G	C	C	G	T	G
JMUB1293	5	II	2149/2151 (99%)		A			A				
JMUB1297	8	IVa	2146/2151 (99%)	T		A	T	A		A		
JMUB1282	8	IVa	2149/2151 (99%)				T	A				
JMUB492	1	IVa	2149/2151 (99%)				T	A				
JMUB1308	89	IVa	2149/2151 (99%)				T	A				
JMUB1311	89	IVa	2149/2151 (99%)				T	A				
JMUB1291	89	IVa	2149/2151 (99%)				T	A				
JMUB1305	91	IVa	2149/2151 (99%)				T	A				
JMUB1979	59	IVa	2149/2151 (99%)				T	A				
JMUB1315	1516	IVc	2148/2151 (99%)	T				A				A
JMUB217	772	V	2148/2151 (99%)	T				A			A	
JMUB1285	89	V	2148/2151 (99%)	T				A			A	
JMUB1289	89	V	2148/2151 (99%)	T				A			A	
JMUB1284	89	V	2148/2151 (99%)	T				A			A	
JMUB1283	89	V	2148/2151 (99%)	T				A			A	
JMUB1304	89	V	2148/2151 (99%)	T				A			A	
JMUB1301	89	V	2148/2151 (99%)	T				A			A	
JMUB1973	59	V	2148/2151 (99%)	T				A			A	
JMUB1981	59	V	2148/2151 (99%)	T				A			A	
JMUB1980	59	V	2148/2151 (99%)	T				A			A	
JMUB1976	59	V	2148/2151 (99%)	T				A			A	
JMUB1978	59	V	2148/2151 (99%)	T				A			A	
JMUB1972	59	V	2148/2151 (99%)	T				A			A	
JMUB1974	59	V	2147/2151 (99%)	T				A	T		A	
JMUB1977	338	V	2148/2151 (99%)	T				A			A	
JMUB1298	121	V	2148/2151 (99%)	T				A			A	
JMUB1303	121	V	2148/2151 (99%)	T				A			A	
JMUB1281	121	V	2148/2151 (99%)	T				A			A	
JMUB1286	121	V	2148/2151 (99%)	T				A			A	
JMUB1300	121	V	2148/2151 (99%)	T				A			A	
JMUB1314	121	V	2148/2151 (99%)	T				A			A	
JMUB1312	121	V	2148/2151 (99%)	T				A			A	
JMUB1313	121	V	2148/2151 (99%)	T				A			A	
JMUB1288	121	V	2148/2151 (99%)	T				A			A	
JMUB1280	121	V	2148/2151 (99%)	T				A			A	
JMUB1295	121	V	2148/2151 (99%)	T				A			A	
JMUB1299	121	V	2148/2151 (99%)	T				A			A	
JMUB1316	121	V	2148/2151 (99%)	T				A			A	
JMUB1294	121	V	2148/2151 (99%)	T				A			A	
JMUB1296	121	V	2148/2151 (99%)	T				A			A	
JMUB1292	121	V	2148/2151 (99%)	T				A			A	
JMUB1310	6217	V	2148/2151 (99%)	T				A			A	
JMUB1302	6217	V	2148/2151 (99%)	T				A			A	

^a^The positions of the nucleotide substitutions refer to sites upstream (−) or downstream (+) of the *mecA* translation start site of N315.

^b^Sequence type of MLST.

Moreover, whole-genome sequencing demonstrated that 34 of the 43 (79%) OS-MRSA isolates carried a complete *bla* operon (Table 1), which could be classified into two genotypes, namely *bla* operon-1 and *bla* operon-2, based on the nucleotide sequences. These two operons shared nucleotide identities of 94% for *blaZ*, 92% for *blaR1*, and 94% for *blaI*. Twelve strains (28%) carried *bla* operon-1, and all but one (JMUB217) *bla* operon-1 was located on plasmids. Meanwhile, 23 isolates (53%) carried intact *bla* operon-2 in their chromosomes. JMUB217 carried both *bla* operons on its chromosome. An incomplete *bla* operon-2 lacking *blaZ* but having intact *blaR1* and *blaI* was present in isolates JMUB1301 and JMUB1313. The absence of *blaZ* in the *bla* operons of these isolates was confirmed by PCR (data not shown). Lastly, seven isolates (16%) lacked a *bla* operon.

### PBP2a production and *mecA* expression in OS-MRSA

**Figure 2:**
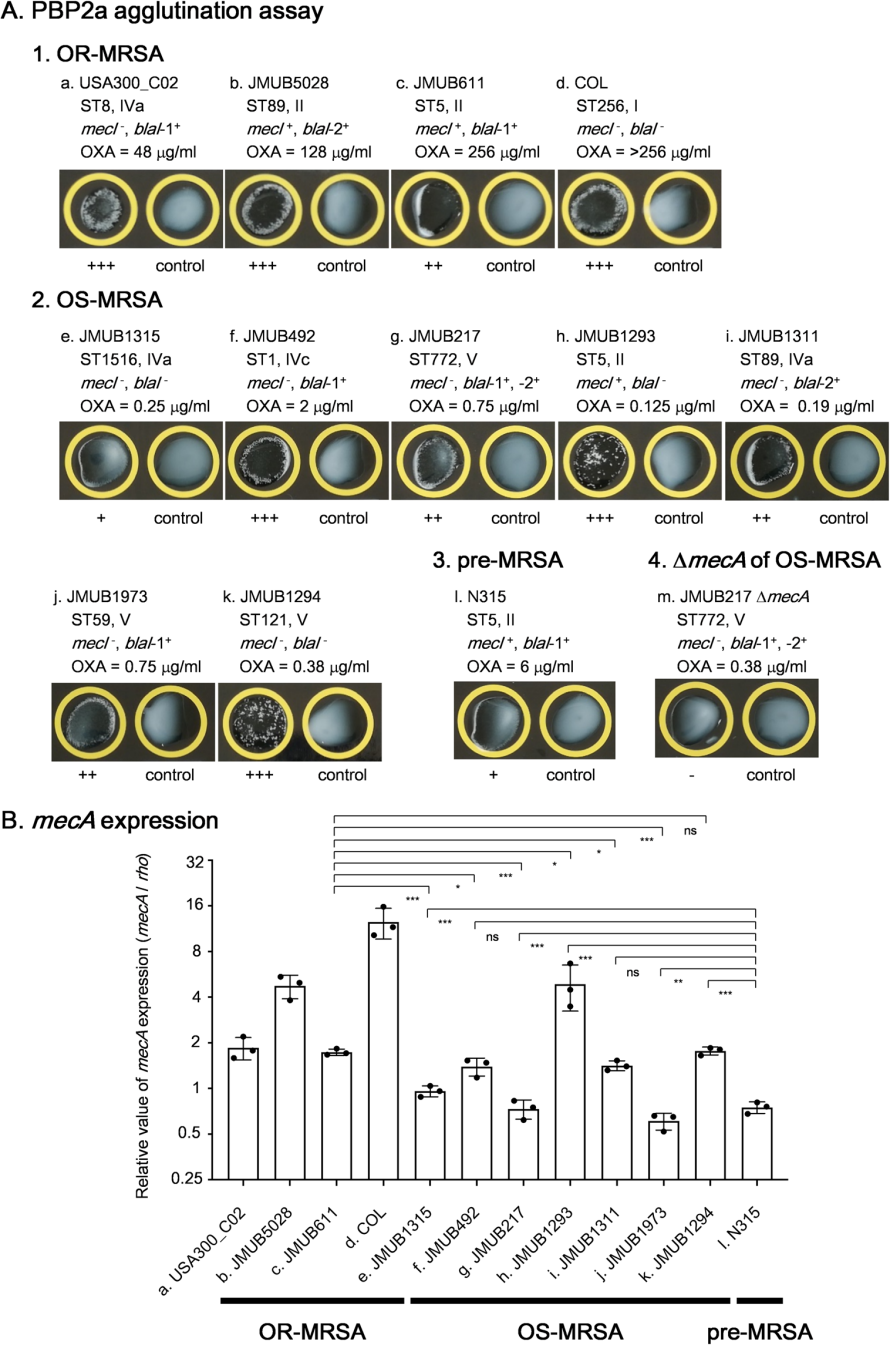
The levels of PBP2a production and *mecA* expression in oxacillin-resistant MRSA (OR-MRSA), oxacillin-susceptible MRSA (OS-MRSA), and pre-MRSA. (**A**) PBP2a agglutination assay results of (1) OR-MRSA, (2) OS-MRSA, (3) pre-MRSA and (4) OS-MRSA JMUB217 ∆*mecA* strain. PBP2a production was detected by the MRSA-screen test (Denka Seiken) based on the agglutination of latex particles sensitized with monoclonal antibodies against PBP2a. (**B**) Quantitation of *mecA* mRNA in OR-, OS- and pre-MRSA by qRT-PCR. The qRT-PCR data are shown as means ± SD of three biological triplicates. *, **, *** and ns indicate *P* < 0.05, 0.01, 0.001 and not significance, respectively by Student’s t-test.

To assess the correlation between oxacillin susceptibility and level of PBP2a production in OS-MRSA, PBP2a agglutination assay (semi-quantitation) was performed using representative OS-MRSA strains of seven different main phylogenetic clades (Fig. 1). Oxacillin-resistant (OR) MRSA strains such as USA300_C02 (ST8, IVa, *mecI*^−^, *blaI*-1^+^, OXA = 48 μg/mL), JMUB5028 (ST89, II, *mecI*^+^, *blaI*-2^+^, OXA = 128 µg/mL), JMUB611 (ST5, II, *mecI*^+^, *blaI*-1^+^, OXA = 256 µg/mL), and COL (ST256, I, *mecI*^−^, *blaI*^−^, OXA > 256 µg/mL) and pre-MRSA strain N315 (ST5, II, *mecI*^+^, *blaI*-1^+^, OXA = 6 µg/mL) were also analyzed as controls. Results showed that all OS-MRSA strains tested produced detectable level of PBP2a (Fig. 2A). JMUB1315 showed weak agglutination reaction similar to pre-MRSA N315, while other strains showed moderate or strong agglutination reactions. In parallel, *mecA* expression level of these strains was also determined by qRT-PCR, and the results were very similar to that of PBP2a semi-quantitation (Fig. 2B). There was no clear correlation seen between the levels of oxacillin MIC and *mecA* expression as well as PBP2a production in OS-MRSA.

### Influence of *bla* operon on reduced susceptibility to oxacillin in OS-MRSA

**Figure 3:**
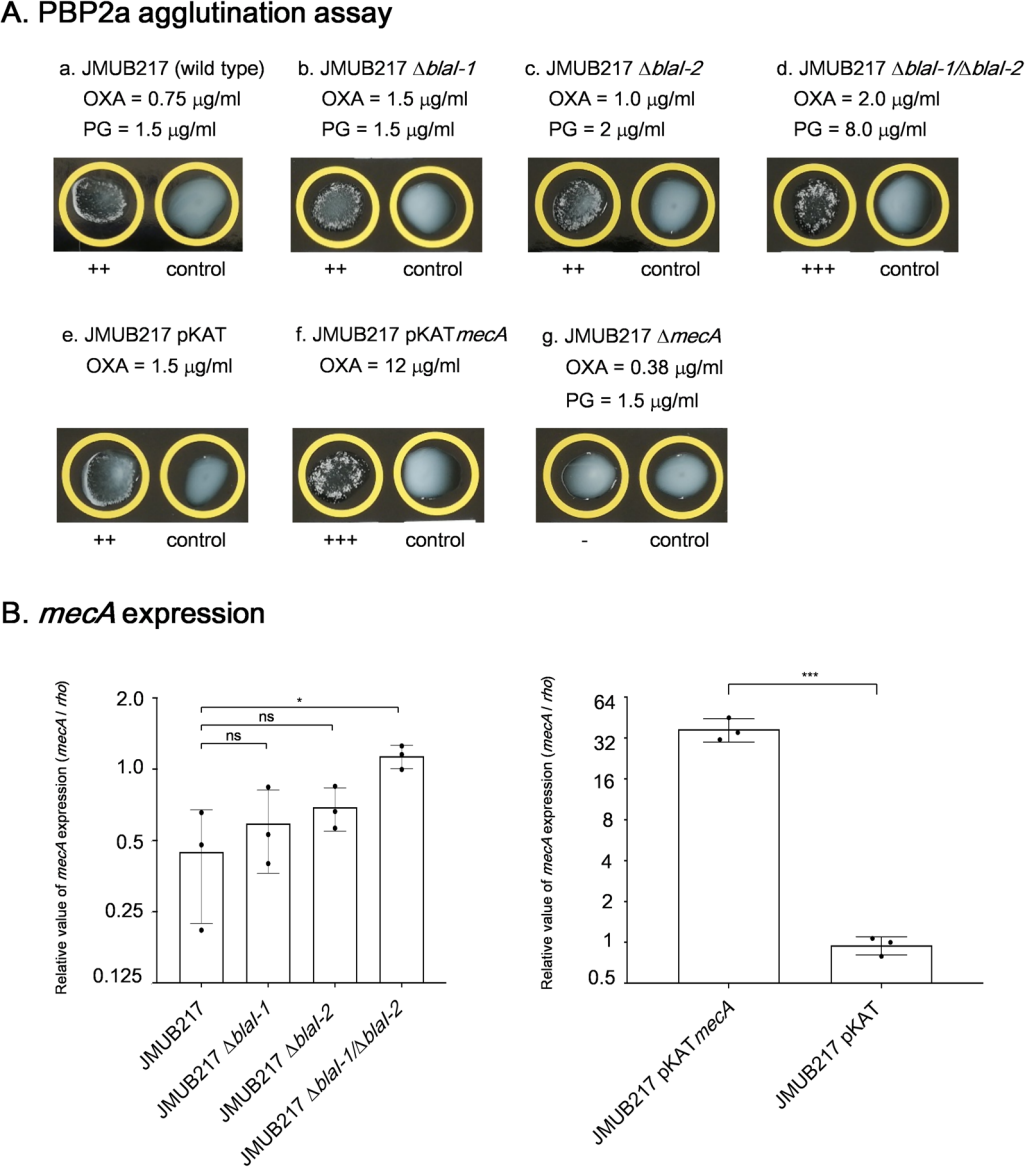
The levels of PBP2a production and *mecA* expression in mutants of *blaI* deletion, *mecA* overexpression and/or *mecA* deletion generated from OS-MRSA strain JMUB217. (**A**) PBP2a agglutination assay results of strains (a) JMUB217, (b) JMUB217 ∆*blaI*-1, (c) JMUB217 ∆*blaI*-2, (d) JMUB217 ∆*blaI*-1/∆*blaI*-2, (e) JMUB217 pKAT (vector), (f) JMUB217 pKAT*mecA*, and (g) JMUB217 ∆*mecA*. PBP2a production was detected by the MRSA-screen test (Denka Seiken) based on the agglutination of latex particles sensitized with monoclonal antibodies against PBP2a. (**B**) qRT-PCR quantitation results of *mecA* mRNA in *blaI* deletion and *mecA* overexpression mutants of JMUB217. The data are shown as means ± SD of three biological replicates. *, **, *** and ns indicate *P* < 0.05, 0.01, 0.001 and not significance, respectively by Student’s t-test.

A previous study suggested that *blaI* expression levels were associated with reduced oxacillin susceptibility in OS-MRSA isolates^26^. To understand how the *bla* operons affect oxacillin susceptibility in the tested OS-MRSA strains, mutants with knockout of β-lactamase repressor gene *blaI* were generated and their effect on the oxacillin susceptibility was analyzed. Our whole-genome sequencing analysis showed that *bla* operon were carried by 36 of the 43 (84%) OS-MRSA isolates, and the *bla* operons could be classified into two genotypes, *bla*-1 and *bla-*2*.* The OS-MRSA JMUB217 (ST772, V, *mecI*^−^, *blaI*-1^+^, *blaI*-2^+^, OXA = 0.75 µg/ml) carried both *blaI*-1 and *blaI-*2, therefore, we deleted either one or both of their *blaI* to generate single and double *blaI*-knockout mutants, and their MICs of penicillin G and oxacillin were determined (Fig. 3A). Knockout of *blaI*-1 or *blaI*-2 alone did not significantly affect MICs of the penicillin G and oxacillin, whereas the double knockout could raise MIC of penicillin G significantly from 1.5 to 8 µg/mL, but of oxacillin slightly from 0.75 to 2 µg/mL. Similar to the results of MIC determination, knockout of *blaI*-1 or *blaI*-2 alone did not affect the levels of *mecA* expressions and PBP2a production, whereas the double knockout enhanced *mecA* expression and PBP2a production but to a lesser extent (Fig. 3B). These results indicated that the influence of *blaI* on oxacillin susceptibility is limited.

### Identification of mutations associated with reduced susceptibility to oxacillin in OS-MRSA

**Figure 4:**
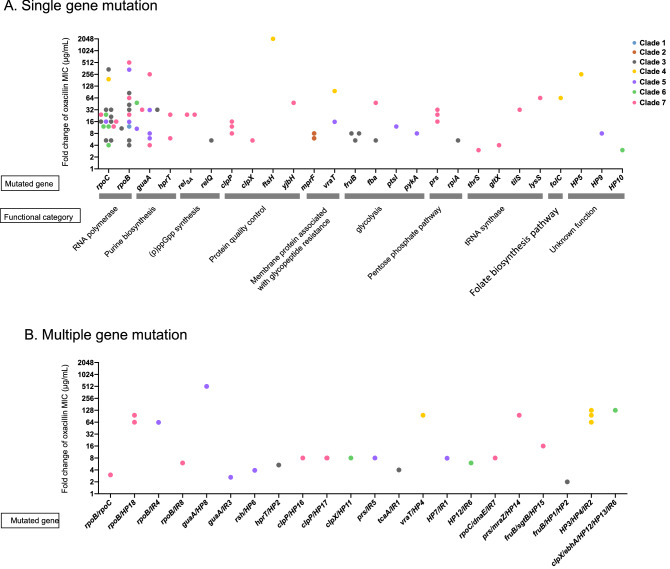
Relationship between gene mutation and fold changes of oxacillin MIC in the mutants with reduced oxacillin susceptibility. Each mutant with reduced oxacillin susceptibility is represented by a closed circle, and different color-coded circles indicate different phylogenetic clades. (**A**) Fold changes of oxacillin MICs for mutants carrying single or double mutations in the same gene. (**B**) Fold changes of oxacillin MICs for mutants carrying single or double mutations in different genes.

To elucidate the pathway(s) leading to the acquisition of β-lactam resistance in OS-MRSA, laboratory-derived mutants with reduced susceptibility to oxacillin were obtained from the parent OS-MRSA strains via single-step exposure to oxacillin. Although resistant colonies growing inside the inhibition zone were generated from all 43 parent strains, not all selected colonies displayed increased oxacillin MICs after single-colony purification. This might be due to the hetero-resistance phenotype of OS-MRSA as some OS-MRSA strains were reported to show heterogeneous resistance to oxacillin^18,22^. Re-determination of the oxacillin susceptibility of the isolated colonies identified 100 mutants with increased oxacillin MICs (ranged from 1 – 256 µg/mL) generated from 26 OS-MRSA strains representing all seven phylogenetic clades (Fig. 1). Among them, 86 mutants exhibited MICs of greater than 4 µg/mL (Table S2). The comparative genomic analysis of the 100 mutants and their respective parent OS-MRSA strains identified a total of 141 mutations, and all mutations were verified via Sanger sequencing (Table S2). It was found that 70 out of 100 mutants carried only one mutation, four mutants carried two mutations in a single gene, and 26 mutants carried multiple mutations in different gene or intergenic region. Moreover, 96 mutants carried at least one nonsynonymous or frameshift mutation, and 4 mutants (JMUB1283-3, JMUB1972-1, JMUB1281-7, and JMUB1310-6) had silent mutations (HP7^T450C^, HP10^C651T^, *guaA*^G1158A^*,* and *tilS*^A1287C^; Table S2). Of the 129 mutations identified in coding sequences, 98 (76%), 13 (10%), 10 (7.8%), and 8 (6.2%) were missense, nonsense, frameshift, and synonymous mutations, respectively. Among them, 121 nonsynonymous mutations were distributed within 46 genes (Table S2; Fig. 4), but no mutation was found in *mec* or *bla* operons. The 46 mutated genes could be classified into 13 functional categories: (1) DNA/RNA polymerase, *rpoC* (22 mutations), *rpoB* (20 mutations), and *dnaE* (one mutation); (2) purine biosynthesis, *guaA* (nine mutations), and *hprT* (three mutations); (3) (p)ppGpp synthase, *rel*_*Sau*_ (four mutations), and *relQ* (one mutation); (4) protein quality control, *clpP* (six mutations), *clpX* (three mutations), *ftsH* (one mutation), and *yjbH* (one mutation); (5) membrane protein associated with glycopeptide resistance, *mprF* (four mutations), *tcaA* (one mutation), and *vraT* (three mutations); (6) glycolysis, *fruB* (five mutations), *fbaA* (two mutations), *ptsI* (one mutation), and *pykA* (one mutation); (7) pentose phosphate pathway, *rpiA* (one mutation) and *prs* (five mutations); (8) tRNA synthesis, *thrS* (one mutation), *tilS* (one mutation), *gltX* (one mutation), and *lysS* (one mutation); (9) folate biosynthesis, *folC* (one mutation); (10) peptidoglycan biosynthesis, *sgtB* (one mutation); (11) transcriptional regulation, *mraZ* (one mutation); (12) extracellular matrix protein, *ebhA* (one mutation); and (13) unknown function, HP1-HP18 (27 mutations). On the other hand, the 12 mutations identified in eight intergenic regions were located between genes of SA0499 and *rpoB*, *sgtB* and SA1692, E8M03_00305 and *hsdR*, SA2092 and *ssaA2*, SAS044 and SA1196, *norB* and *ebhA*, *tnp* and *proP*, and SA1447 and *alaS*, respectively. These results clearly demonstrated that mutations responsible for oxacillin resistance in the OS-MRSA-derived mutants are quite diversified.

### Contribution of increased *mecA* expression to reduced oxacillin susceptibility in OS-MRSA

To understand the role of the identified mutations in reduced oxacillin susceptibility, OS-MRSA strain JMUB217 and its oxacillin-resistant mutants were used as representative strains for further study because 24 mutants carrying 26 variants in 11 genes and an intergenic region were derived from the JMUB217. In addition, oxacillin MICs for the 24 mutants ranged widely (1.5–256 µg/mL), and were highly elevated compared to their parent strain JMUB217 (0.75 µg/mL). A sequential experiment was carried out. First, a *mecA*-overexpressing mutant was created to investigate whether changes in *mecA* expression affects oxacillin susceptibility in OS-MRSA. A vector pKAT containing *mecA* and its native promoter was introduced into JMUB217 to generate the *mecA*-overexpressing mutant JMUB217 (p*mecA*), and MIC determination found that the generated mutant exhibited increment of oxacillin MIC from 0.75 to 12 µg/mL (Fig. 3; Table 3). Next, *mecA*-knockout mutant JMUB217 (∆*mecA*) was generated and its oxacillin MIC was measured. The oxacillin MIC decreased slightly from 0.75 to 0.38 µg/mL in this *mecA*-deleted mutant (Table 3), indicating that the presence of *mecA* itself confers a low level oxacillin resistance. Moreover, overexpression of *mecA* in the *mecA*-deleted mutant JMUB217 (∆*mecA*) resulted in an increment of the oxacillin MIC to 12 µg/mL, similar to that of the *mecA*-overexpressing mutant JMUB217 (p*mecA*). Finally, a set of *mecA*-knockout strains from three oxacillin-resistant mutants (JMUB217-11, JMUB217-22, and JMUB217-24), carrying mutations of RpoC^P358L^, RpoB^G645H^, and RpoC^G498D^, respectively, were generated and their oxacillin MICs were determined. The results found that their MICs of 4, 32, and 256 µg/mL decreased to 0.38 µg/mL, similar to the level of the *mecA*-knockout mutant JMUB217 (∆*mecA*) (Table 3). These results indicated that *mecA* expression is a key factor for promoting reduced oxacillin susceptibility in OS-MRSA.

**Table 3:** Oxacillin MIC of *mecA* overexpression and deletion derivatives in OS-MRSA JMUB217 and its oxacillin-reduced susceptibility mutants.

Strain	Description	Oxacillin MIC (µg/mL)
JMUB217	Wild type	0.75
	pKAT	1.5
	pKAT-*mecA*	12
	*∆mecA*	0.38
	*∆mecA*-pKAT-*mecA*	12
JMUB217-11	RpoC^P358L^	4
	RpoC^P358L^-∆*mecA*	0.38
JMUB217-22	RpoB^Q645H^	32
	RpoB^*Q645H*^*-∆mecA*	0.38
JMUB217-24	RpoC^G498D^	256
	RpoC^G498D^*-∆mecA*	0.38

### Correlation of the levels of *mecA* expression and PBP2a production with oxacillin MIC in mutants with reduced oxacillin susceptibility

**Figure 5:**
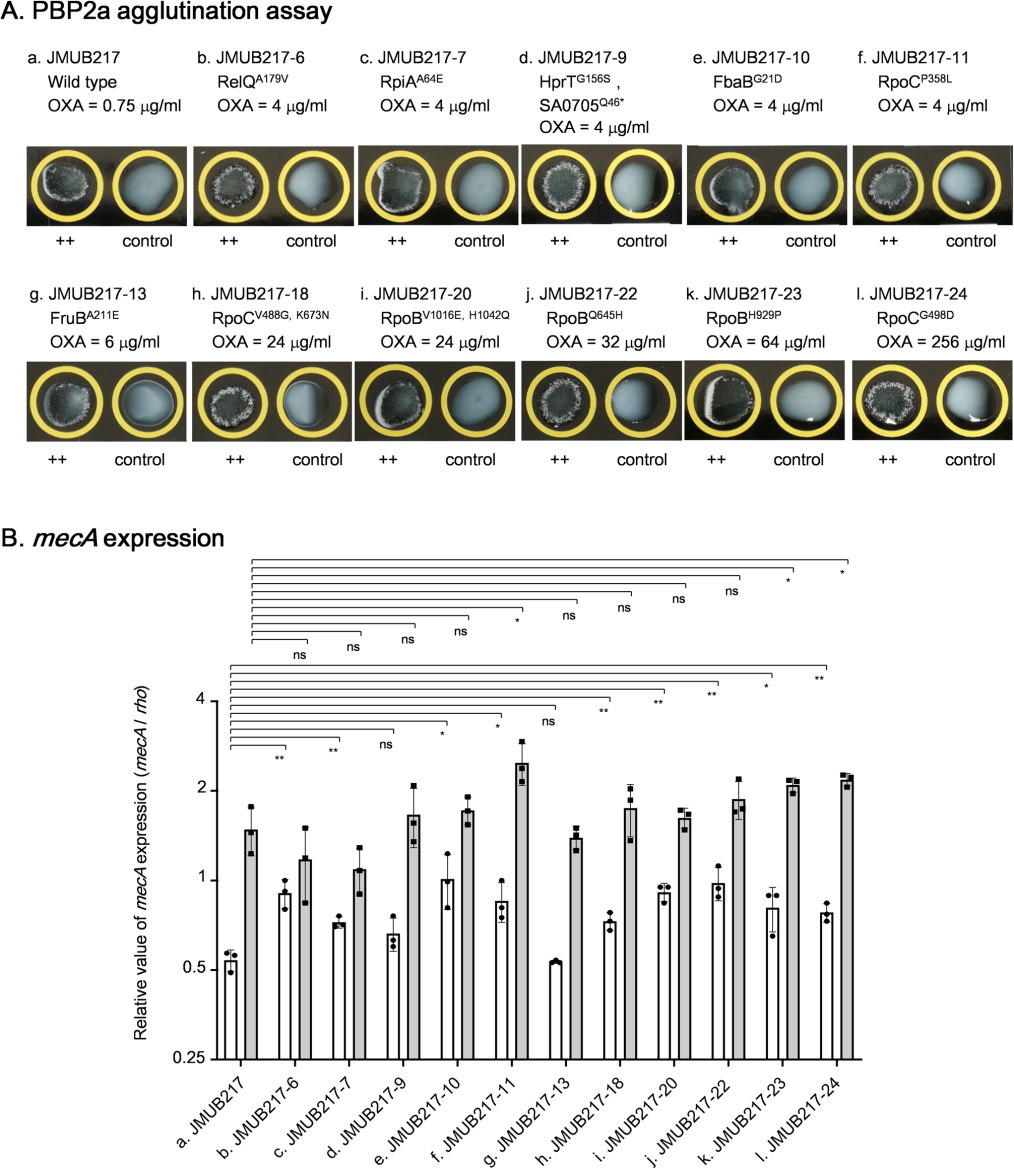
The levels of PBP2a production and *mecA* expression in JMUB217-derived mutants with reduced oxacillin susceptibility. (**A**) PBP2a agglutination assay of mutants with reduced oxacillin susceptibility. PBP2a production was detected by the MRSA-screen test (Denka Seiken) based on the agglutination of latex particles sensitized with monoclonal antibodies against PBP2a. (**B**) qRT-PCR quantitation of *mecA* mRNA in mutants with reduced oxacillin susceptibility under the absence (white bars) and the presence (grey bars) of oxacillin induction. The qRT-PCR data are shown as means ± SD from biological triplicates. *, **, *** and ns indicate *P* < 0.05, 0.01, 0.001 and not significance, respectively by Student’s t-test.

To examine the correlation between PBP2a production and oxacillin susceptibility in laboratory mutants, PBP2a agglutination assay was performed on 11 JMUB217-derived mutants (Fig. 5A). Despite the increase of oxacillin MIC, PBP2a production was not significantly changed in these mutants. Next, *mecA* expression of JMUB217-derived mutants in the presence and absence of oxacillin was measured (Fig. 5B). The results showed that the natural expression of *mecA* significantly increased in 9 of 11 mutants with 1.3 to 1.9-fold change. However, the *mecA* expression levels were still lower than those of OR-MRSA (Fig. 2B). Since *bla* operons of JMUB217 induced *mecA* expression (Fig. 3), the *mecA* expression levels were measured in the presence of low concentration of oxacillin (0.1 µg/mL). Results showed that oxacillin induction significantly upregulated the *mecA* expression level in wild-type (2.8-fold) as well as the resistant mutants (1.3 to 2.9-fold). When compared with wild-type strain, the *mecA* expression levels induced by oxacillin were significantly increased in three of 11 mutants (JMUB217-11, -23, -24). Interestingly, the resistant mutant with the highest oxacillin MIC did not display the strongest *mecA* expression in both presence and absence of oxacillin. As seen in Fig. 5B, JMUB217-24 had the highest oxacillin MIC of 256 µg/mL, but showed a similar *mecA* expression level compared to the mutants JMUB217-23, JMUB217-22, JMUB217-18, JMUB217-11 and JMUB217-9, which had oxacillin MICs of 64, 32, 24, 4, and 4 µg/mL, respectively (Table S2; Fig. 5), indicating that *mecA* expression was not the only cause of oxacillin resistance in the mutants.

### Transcriptome analysis revealed a stringent-like response in the oxacillin-resistant mutants

To figure out the overall gene regulation in the oxacillin-resistant mutants, which alters transcriptional profile and ultimately biases gene regulation toward the expression of oxacillin resistance, the whole-genome expression profiles of five representative mutants of JMUB217, JMUB217-7 carrying RpiA^A64E^ (oxacillin MIC = 4 µg/mL), JMUB217-11 carrying RpoC^P358L^ (oxacillin MIC = 4 µg/mL), JMUB217-18 carrying RpoC^V488G,K673N^ (oxacillin MIC = 24 µg/mL), JMUB217-20 carrying RpoB^V1016E,H1042Q^ (oxacillin MIC = 24 µg/mL), and JMUB217-22 carrying RpoB^Q645H^ (oxacillin MIC = 32 µg/mL), were analyzed and compared with that of the parent strain JMUB217. The transcriptome analyses were performed under both oxacillin-induced and drug-free growth conditions. Differentially expressed genes (DEGs) with log_2_-fold change of >  ±1 (adjusted *P* value of < 0.01) were identified by pair-wise comparison (Table S3). In concordance with qRT-PCR data (Fig. 5B), the results of transcriptome analysis showed that *mecA* expression was significantly induced by oxacillin in both the mutants and parent strain, while the differences in the basal *mecA* expression between the mutants and its wild-type was small (log_2_-fold change < 1) as shown in Table S3.

**Figure 6:**
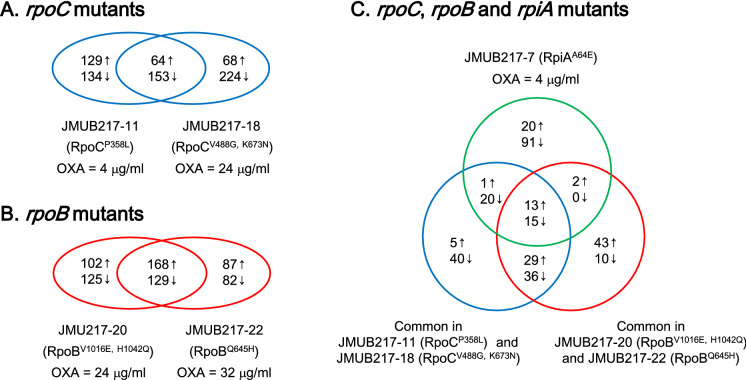
Venn diagram summary of differently expressed genes identified among the five representative mutants from OS-MRSA strain JMUB217. The number of genes co-upregulated and -downregulated with log_2_-fold change of > 1 (*P* value of < 0.01) in the mutants with mutation of (**A**) *rpoC*, (**B**) *rpoB* , and (**C**) *rpoC*, *rpoB,* and *rpiA ,* respectively, are listed.

**Figure 7:**
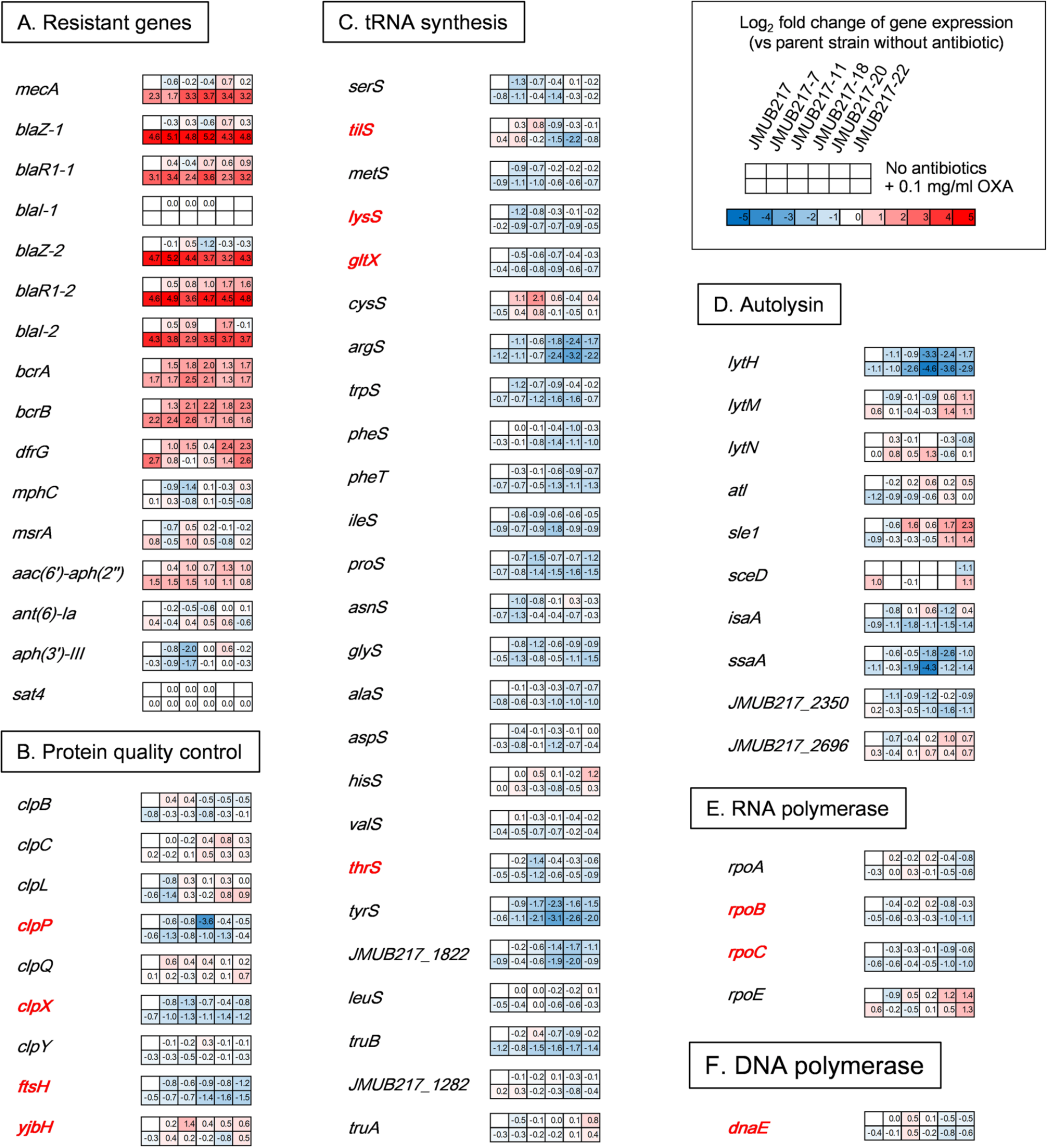

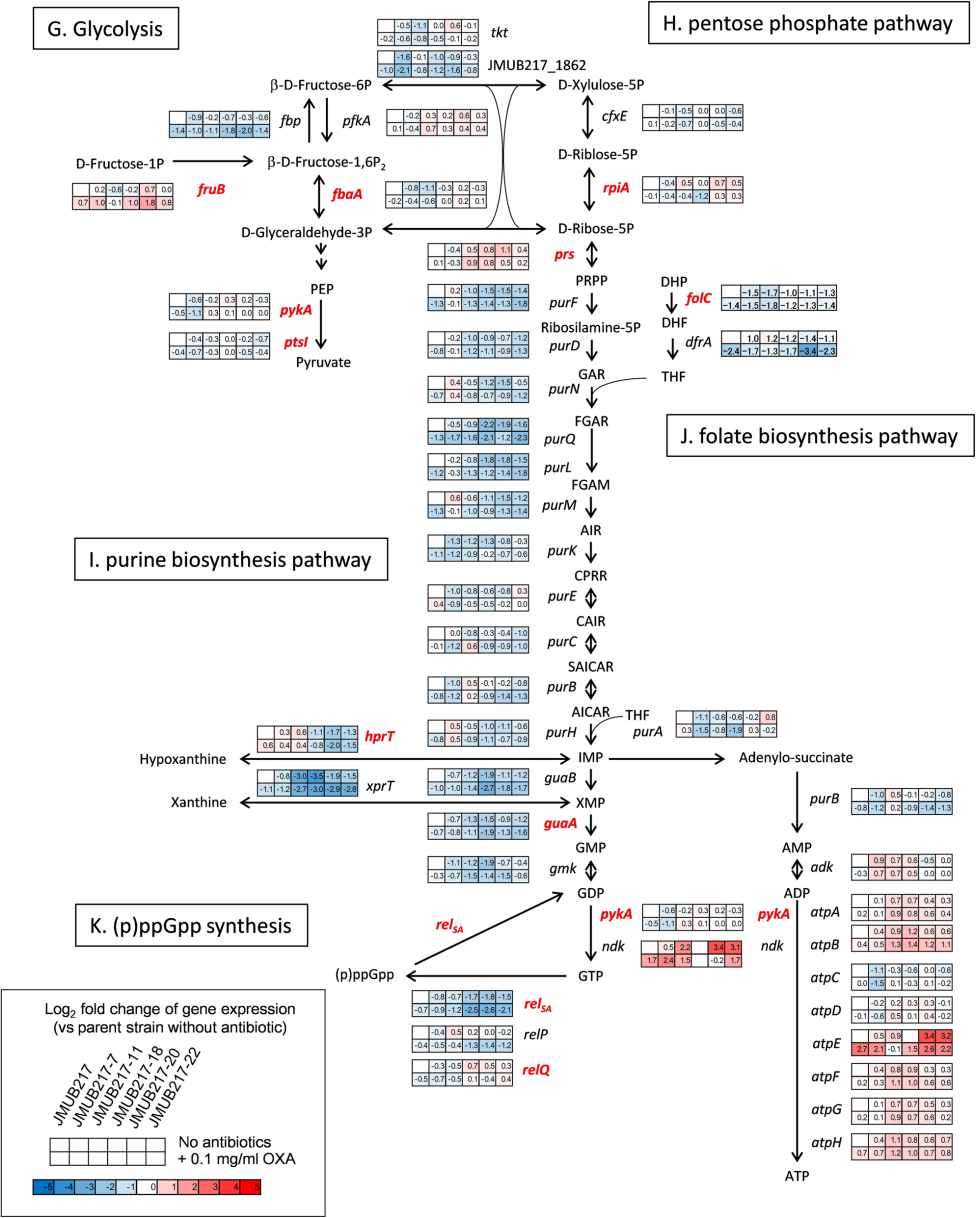

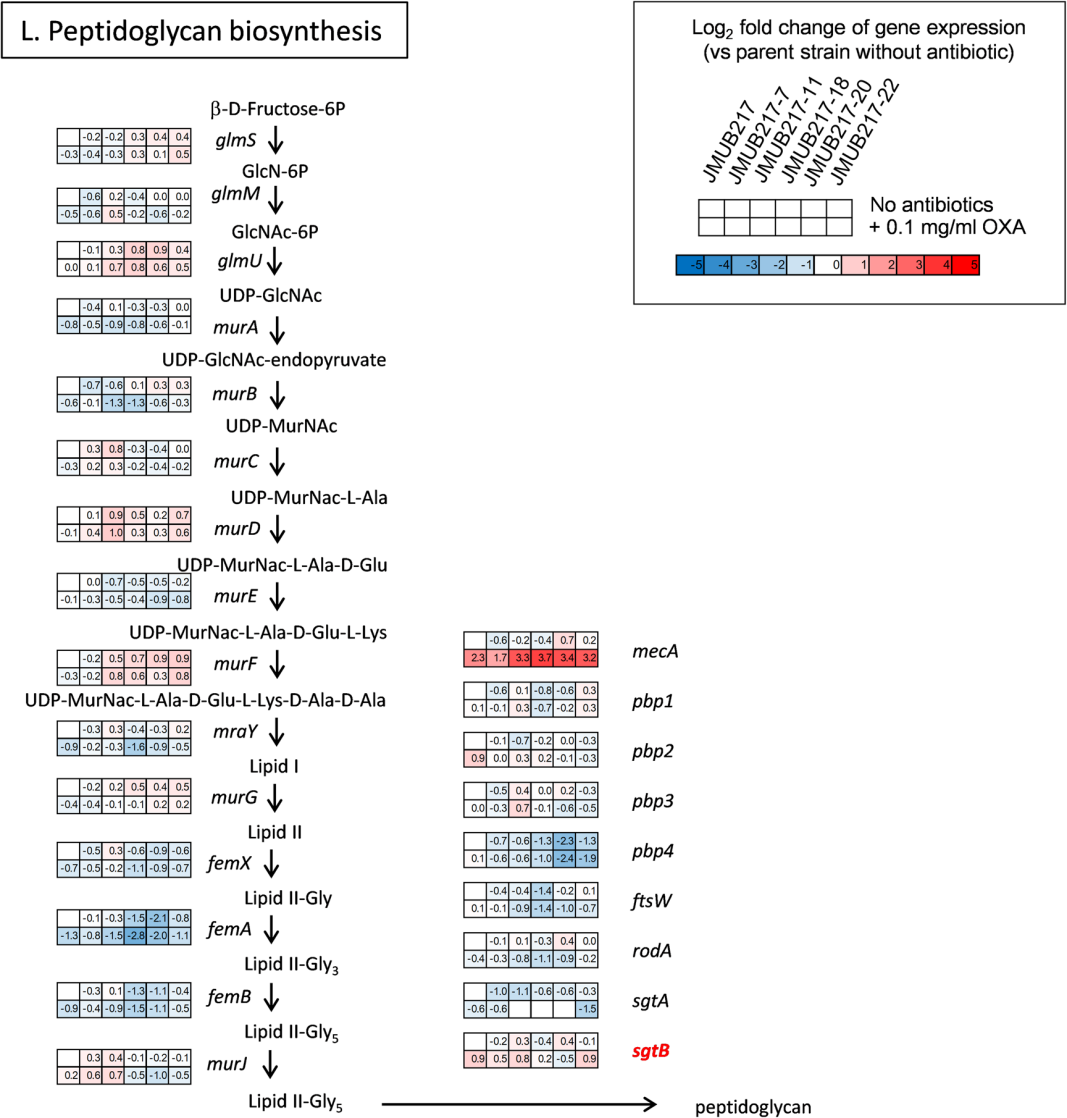
Gene expression profiles of OS-MRSA strain JMUB217 and its mutants with reduced susceptibility to oxacillin in the presence and absence of oxacillin induction. Differentially expressed genes between the parent strain JMUB217 and its mutants were classified into 12 different functional categories, including (**A**) antibiotic resistance, (**B**) protein quality control, (**C**) tRNA synthesis, (**D**) autolysis, (**E**) RNA polymerase activity, (**F**) DNA polymerase activity, (**G**) glycolysis, (**H**) the pentose phosphate pathway, (**I**) purine biosynthesis, (**J**) folate biosynthesis, (**K**) (p)ppGpp synthesis, and (**L**) peptidoglycan biosynthesis. The color scales indicate the degree of log_2_-fold changes of transcriptional expression in the mutants compared with that in wild-type strains without induction. Mutated genes identified in the mutants with reduced oxacillin susceptibility are shown in red font.

A Venn diagram analysis showed that two *rpoC* mutants JMUB217-11 and JMUB217-18 shared 217 DEGs, of which 64 or 153 genes were commonly upregulated or downregulated, respectively (Fig. 6A). Two *rpoB* mutants JMUB217-20 and JMUB217-22 shared 297 DEGs with 168 up- and 129 down-regulated genes (Fig. 6B). In case of all five strains carrying mutation of either *rpiA or rpoC* or *rpoB*, 13 genes were up- and 15 genes down-regulated commonly (Fig. 6C; Table S4). Among the commonly regulated genes, upregulation of tryptophan biosynthesis genes (*trpBDEFG*) and downregulation of nucleotide transporter and biosynthesis genes (*pyrRP, hisIG*). These genes were known to be related with Rel_Sau_/RSH-dependent stringent response mediated by amino-acid deprivation in *S. aureus*^27^. Rel_Sau_ is a bifunctional (p)ppGpp synthase/hydrolase and induces the classic stringent response by accumulation of (p)ppGpp^28^. In addition, downregulation of purine biosynthesis genes such as *xprT*, *purF* and *guaB*, which were usually seen in the stringent response were observed in the four *rpoC* and *rpoB* mutants (Table S4; Fig. 7I).

In addition to the alteration of expression of genes directly related to the stringent response, the mutants with reduced oxacillin susceptibility also exhibited downregulation of genes involved in protein quality control and tRNA synthesis. Notably, *clpP*, *clpX*, and *ftsH* were significantly downregulated in mutants with reduced oxacillin susceptibility (Fig. 7B). Moreover, 16 out of 25 tRNA genes were also downregulated in at least one of the five mutants (Fig. 7C). These changes in gene expression might contribute to oxacillin resistance, as previous studies described that deficiencies of protein quality control and tRNA synthesis were associated with the stringent response and β-lactam resistance^29–31^.

Transcriptome profiles of the *rpoBC* and *rpiA* mutants also showed alteration in gene expression relevant to the peptidoglycan biosynthesis, for example, upregulation of *mecA* and *sgtB,* and downregulation of *murBJY*, *femABX*, *pbp4*, *ftsW*, and *rodA* (Fig. 7L). Furthermore, changes in the expression of genes involved in autolysis of *S. aureus* were observed in strains carrying mutations of *rpoBC* and *rpiA,* for example, *lytM* and *sle1* were upregulated, whereas *lytH*, *isaA*, and *ssaA* were downregulated (Fig. 7D). All these changes in combination with *mecA* expression and stringent-like response might direct bacterial metabolism towards acquisition of oxacillin resistance in the resistant mutants.

### The mutants with reduced oxacillin susceptibility tend to slow growth

Mutations in genes involved in the stringent response were reported to be associated with slower growth rate^32^. In addition, some β-lactam–resistant mutants generated in vitro were also found to have slow growth rates or a phenotype of persistent infection^31–34^. To investigate whether the mutations identified in the oxacillin-resistant mutants affect cell growth, the doubling time of JMUB217-derived mutants was measured. Seven of eleven mutants showed significantly slower growth speed (doubling time 36.5 ± 0.7 min to 57.7 ± 1.1 min) compared to their parent strain (32.7 ± 1.4 min). In contrast, three of them had similar doubling time (JMUB217-11, 34.1 ± 0.3 min; JMUB217-24, 34.8 ± 0.4 min; JMUB217-9, 35.3 ± 0.9 min) and JMUB217-7 grew faster than wild type (29.0 ± 0.3 min). Surprisingly, growth of the JMUB217-21 carrying GuaA^I249fs^ mutation was very slow (347.1 ± 9.3 min).

### Intracellular ATP level in the mutants with reduced oxacillin susceptibility

**Figure 8:**
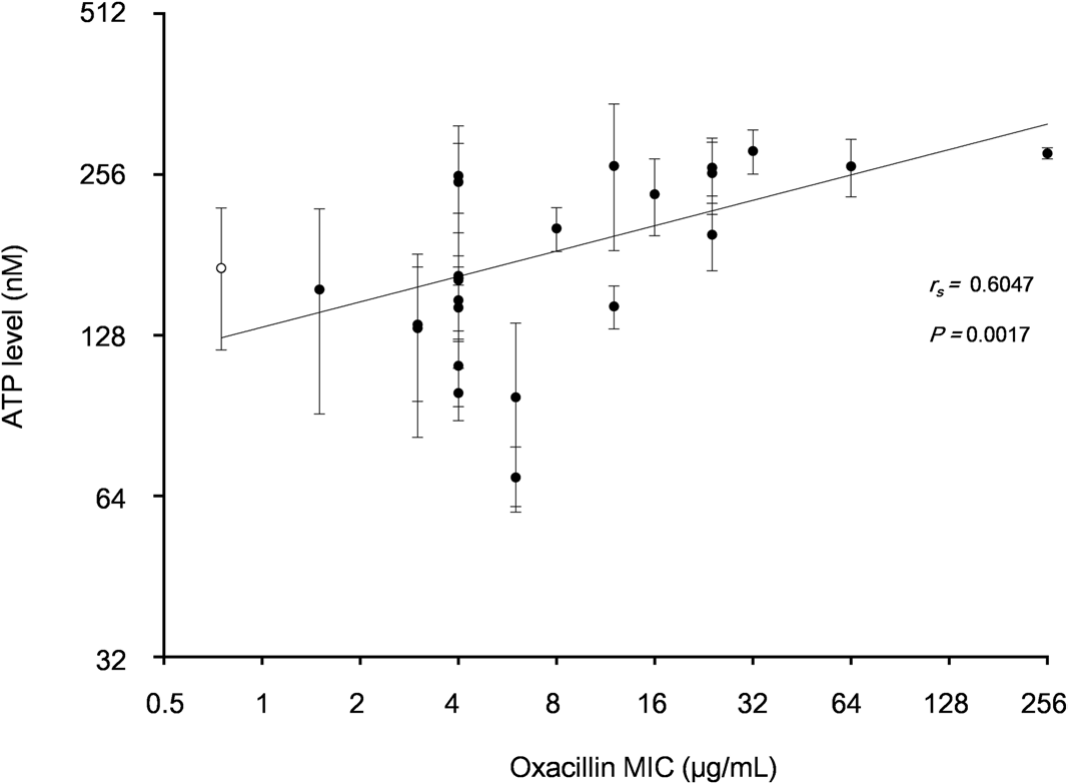
Correlation between the levels of intracellular ATP and oxacillin MIC among the OS-MRSA strain JMUB217 and its mutants with reduced susceptibility to oxacillin. The levels of intracellular ATP are shown as means ± SD of three biological replicates. The correlation coefficient was evaluated using Spearman’s correlation coefficient test. Open circle denotes parent strain and closed circle does the mutants.

Some reports examining intracellular ATP levels of *S. aureus* in relation to stress responses found that lower cellular ATP production was associated with bacterial tolerance to several environmental stresses such as salt, cold, and antibiotics, and it could also induce the conversion of bacterial cells into persistent forms, including small colony variants^35,36^. Our transcriptomics study with the resistant mutants illustrated that several genes involved in purine biosynthesis and folate biosynthesis were clearly downregulated (Fig. 7I,J), which was similar to the findings of Cassels et al.^37^. However, some genes involved in ATP biosynthesis were significantly upregulated (Fig. 7J). To analyze whether the mutations of oxacillin-resistant mutants affect ATP biosynthesis, the intracellular ATP levels of 23 mutants and their parent strain JMUB217 were measured. The results showed that the intracellular ATP was increased in the mutants compared to the parent strain, and there was good correlation between the levels of intracellular ATP and oxacillin MIC with correlation coefficient of 0.6047 (*p* = 0.0017) (Fig. 8).

## Discussion

Since its first description in 1991, OS-MRSA^15,38^, which is related to borderline methicillin-resistant MRSA^39^, has been frequently isolated in hospital and community settings with a prevalence rate of 0.62 – 33.7%^12,15,18,20,40,41^. The presence of OS-MRSA is currently a challenge in the clinical management of staphylococcal infections and requires great attention because it is prone to be misidentified as MSSA via routine β-lactam susceptibility testing^10,12,14^. Indeed, the majority of the OS-MRSA isolates used in this study were initially identified as MSSA according to the oxacillin susceptibility profile provided by the original laboratory despite carrying *mecA*. Similarly, susceptibility testing using cefoxitin, a stronger inducer of the *mecA* regulatory system than oxacillin that is used to detect methicillin resistance^42^, failed to accurately identify OS-MRSA (Table 1). These observations suggest that a combination of oxacillin and cefoxitin susceptibility tests, as recommended elsewhere^43^, or detection of *mecA* will be more reliable for the identification of MRSA.

Despite being phenotypically susceptible to oxacillin, β-lactam resistance can easily be induced in OS-MRSA^20,21,44^. The mechanisms regulating oxacillin susceptibility in *S. aureus* appear to differ depending on the types of mutations and genetic basis of the individual isolates. Chen et al. reported that mutations in the MecI-binding site of the *mecA* promoter downregulated the expression of PBP2a and increased the susceptibility of ST59-SCC*mec* type V strains to oxacillin^45^. Meanwhile, they demonstrated that mutation of the ribosome-binding site of *mecA* in an ST59-SCC*mec* type IV strain attenuated its oxacillin resistance. Nevertheless, these mutations affect only oxacillin resistance in the strains of ST59 background, whereas mutations in the same locus barely affected the β-lactam resistance levels of isolates with different genetic backgrounds, such as COL (ST250) and CH482 (ST45)^24^. Conversely, mutations in the *mecA* coding region conferred oxacillin resistance to OS-MRSA strains isolated in the US^21^. These studies suggested that the inclusion of OS-MRSA strains with a diverse genetic backgrounds is crucial for providing a comprehensive insight into understanding the mechanism of oxacillin resistance in OS-MRSA.

Although oxacillin resistance in OS-MRSA might be caused by increased *mecA* expression, the exact mechanism triggering *mecA* overexpression is unknown. The structure of *bla* operon is highly homologous to the *mec* operon^46^, and quite a high portion (84%) of OS-MRSA isolates analyzed in this study carried *bla* operon (Table 1; Fig. 1), which suggest that *bla* operon might influence oxacillin susceptibility in OS-MRSA. However, deletion of the repressor gene *blaI* from an OS-MRSA strain JMUB217 resulted in only slight increases of the oxacillin MIC. In addition, *mecA* levels were not uniformly increased in a set of JUMB217-derived oxacillin-resistant mutants compared to the parent strain JUMB217, as determined by qRT-PCR. Therefore, we postulated that oxacillin resistance in OS-MRSA strains involves a more complex regulatory pathway than simply direct *mecA* signaling.

The stringent stress response governed by the alarmone (p)ppGpp is involved in the β-lactam resistance of MRSA^34,47^. Both our whole-genome comparative analysis and RNA-seq analysis demonstrated that many mutations identified in the resistant mutants (Fig. 4) and their altered gene transcriptions (Fig. 7) were associated with depletion of pathway relevant to the purine biosynthesis, protein quality control, and tRNA synthesis, which was very similar to the massive transcription downregulation seen in *S. aureus* during the stringent response. However, the expression profiles of stringent response elements in oxacillin-resistant mutants derived from this study were not indicative of the classic stringent response elicited by mupirocin treatment^48^. During the classic stringent response, the cellular stresses resulting from amino acid starvation and mupirocin exposure induce the accumulation of uncharged (deacylated) tRNA^49^. The uncharged tRNA in turn binds to the A (aminoacyl-tRNA) site of the 70S ribosome and activates Rel_Sau_ to produce (p)ppGpp^50^. However, in some Gram-positive bacteria like *Bacillus subtilis*, (p)ppGpp does not directly regulate RNA polymerase (RNAP). Rather, (p)ppGpp synthesis reduces intracellular GTP levels, subsequently leading to the induction of the stringent response^51,52^. Hence, mutations in genes involved in glycolysis, pentose phosphate biosynthesis, folate synthesis, and purine biosynthesis might mimic the (p)ppGpp-mediated reduction in intracellular GTP levels and induce “stringent-like response”, as evidenced by the downregulation of genes responsible for GTP biosynthesis (purine and folate biosynthesis, pentose phosphate biosynthesis, and glycolysis) (Fig. 7). In addition, gene mutations identified in this study included many stringent response elements, and most of them were previously reported to be associated with conversion of hetero- to homo-resistance against β-lactam, such as genes associated with RNA polymerase (RNAP; *rpoB*^31,53^ and *rpoC*^31,54^), purine biosynthesis (*guaA*^31^, *prs*^31,33^, *hprT*^31,33^, (p)ppGpp synthesis (*rel*_*Sau*_^31,33,47^), glycolysis (*pykA*^31^, *fbaA*^31^, and *fruB*^33^) protein quality control (*ftsH*^31^, *clpX*^30,55^, and *clpP*^30,55^), and tRNA synthase (*lysS*^31^, and *gltX*^31^). These results suggest that stringent-like response played an important role in β-lactam resistance in the oxacillin-resistant mutants of OS-MRSA. It remains to be further studied, however, how this response leads to substantial metabolic changes towards acquisition of the resistance.

OS-MRSA is considered problematic in the clinical setting because the strain is prone to develop high-level β-lactam resistance during the course of antibiotic treatment^20,44^. Because the targets of antibiotics are generally essential proteins in bacteria, the acquisition of antibiotic resistance is usually associated with a fitness cost^56^. In *S. aureus*, slow-growth phenotypes, including the formation of small colony variants, are associated with tolerance to antibiotics^57–60^. Contrarily, some of the mutations identified in the JMUB217 strain altered its oxacillin susceptibility without affecting its doubling time. This suggested that the mutations conferring reduced oxacillin susceptibility in OS-MRSA may incur only small fitness costs because of the complementary upregulation of ATP synthase genes. The increased expression of ATP biosynthesis genes was supported by the positive correlation between oxacillin MICs and intracellular ATP levels (Fig. 8), which might explain the easy acquisition of oxacillin resistance in OS-MRSA. Nonetheless, chromosomal mutations in *rpoBC* and other genes involved in purine biosynthesis were identified in slow VISA strains^61,62^, indicating that the fitness cost of mutations may depend on the genetic background of individual strains.

This study aimed to understand the genetic pathways associated with oxacillin resistance in OS-MRSA isolates from diverse genetic backgrounds. Our results suggested that OS-MRSA was rendered oxacillin-resistant by a combination effect of stringent-like response (a stress response similar to the classic stringent response) and subsequent expression of antibiotic resistance genes (e.g., *mecA*, *bla* operon). The relatively low fitness cost of the mutations may fuel the easy selection of oxacillin-resistant OS-MRSA mutants during the course of antimicrobial treatment.

## Materials and methods

### Bacterial strains and growth conditions

A total of 43 OS-MRSA isolates from various clinical samples were collected from routine clinical laboratories in hospitals across Japan and Taiwan between 1998 and 2015 (Table S1^63–65^). Mueller–Hinton broth (MHB; Becton Dickinson Co., Ltd., Sparks, MD, USA) and tryptic soy broth (TSB; Becton Dickinson) were used to culture *S. aureus*, whereas *Escherichia coli* was grown in Luria–Bertani (LB; Becton Dickinson) medium. In some experiments, antibiotics were added to the medium at the following concentrations: ampicillin (Nacalai Tesque, Inc., Kyoto, Japan) at 100 µg/mL for *E. coli*, tetracycline (Nacalai Tesque) at 5 µg/mL for *S. aureus*, and chloramphenicol (Nacalai Tesque) at 10 µg/mL for *S. aureus* and *E. coli*. For preservation, bacterial cells were cultivated on tryptic soy agar (TSA; Becton Dickinson) and incubated at 37 °C upon receipt. A single colony was then selected and grown overnight in TSB at 37 °C. The overnight culture was aliquoted and stored at − 80 °C in 50% glycerol (Wako Pure Chemical Industries, Ltd., Tokyo, Japan) until use.

### Antibiotic susceptibility test

Oxacillin and cefoxitin MICs were determined using the E-test method according to Clinical and Laboratory Standards Institute (CLSI) guidelines^16^. Briefly, overnight cultures of *S. aureus* strains grown in 4 mL of MHB at 37 °C were adjusted to 0.5 McFarland turbidity (approximately 1 × 10^8^ to 2 × 10^8^ CFU/mL) and spread on Mueller–Hinton agar (MHA; Becton Dickinson) plates. The E-test gradient strip (bioMérieux SA, Marcy l’Étoile, France) was then placed on the bacterial lawn. The MIC was determined after incubation at 37 °C for 24 h. The isolates with oxacillin MIC ≤ 2 µg/mL or cefoxitin MIC ≤ 4 µg/mL were considered oxacillin- and cefoxitin-susceptible, respectively.

### *mecA* detection via PCR

DNA was extracted from OS-MRSA isolates grown overnight on TSA plates using MightyPrep reagent (Takara Bio Inc., Shiga, Japan) in accordance with the manufacturer’s instructions. PCR was then performed on the extracted DNA using Quick Taq HS DyeMix (Toyobo Co., Ltd., Osaka, Japan). A primer pair (*mecA*-F and *mecA*-R, Table S5) was used to amplify a 519-bp region of *mecA*. The thermal cycling conditions included initial denaturation at 94 °C for 2 min followed by 30 cycles of 94 °C for 30 s, 55 °C for 30 s, and 68 °C for 1 min. Finally, the amplified products were electrophoresed on 1% agarose gel, stained with ethidium bromide, and visualized using AE-6933FXES Printgraph (Atto Co., Tokyo, Japan).

### PBP2a production

PBP2a was extracted from colonies grow on MHA and was detected using the MRSA-screen latex agglutination test (Denka, Seiken Co. Ltd., Tokyo, Japan) according to the manufacturer’s instructions.

### Isolation of mutants with reduced susceptibility to oxacillin from parent OS-MRSA strains

To isolate mutants with reduced oxacillin susceptibility, all 43 OS-MRSA parent strains were exposed to oxacillin according to the E-test method as described for susceptibility testing. Briefly, the oxacillin E-test was performed on OS-MRSA strains inoculated onto MHA plates. A single colony growing inside the inhibition zone after 24–48 h of incubation was randomly picked and sub-cultured in TSB for 24 h at 37 °C. The overnight culture was then serially diluted tenfold with 0.9% NaCl and spread onto a TSA plate. A single colony growing on the TSA plate was again randomly selected and inoculated into TSB for preservation in 50% glycerol at − 80 °C. The oxacillin susceptibility of the stocked cells was determined again using the E-test method to discriminate mutant colonies from persister colonies. The cells exhibiting higher oxacillin MICs were selected as oxacillin-reduced susceptibility mutants, which were then used for subsequent analysis.

### Whole-genome sequencing

Genomic DNA was extracted from OS-MRSA and its mutants using the phenol–chloroform method and purified using a QIAamp DNA mini kit (Qiagen, Hilden, Germany) following previously developed methods^66^. The genomic sequences of parent strains were determined via mate-pair sequencing as previously described^66,67^. Briefly, a mate-pair library was prepared using a Nextera mate-pair library prep kit (Illumina, Inc., San Diego, CA, USA), and sequencing was performed using an Illumina MiSeq platform with the MiSeq reagent kit version 3 (Illumina). The mate-paired reads of OS-MRSA were trimmed using the FASTQ toolkit version 2.2.0 to generate high-quality reads and assembled using Velvet Assembler version 1.2.10 to construct genome scaffolds. The generated genomic sequences were finally annotated using Microbial Genome Annotation Pipeline (https://www.migap.org/). Meanwhile, the genomic sequences of in vitro-selected mutants with reduced oxacillin susceptibility were determined by sequencing paired-end reads as previously described^68^. The paired-end library was prepared using a Nextera XT library prep kit and sequenced using the Illumina MiSeq platform with the MiSeq reagent kit version 3. The paired-end reads of each mutant were mapped against the genomic sequences of their corresponding parent OS-MRSA strains, and mutations were detected using CLC Genomics Workbench version 9 (CLCbio, Qiagen, Valencia, CA, USA). Mutations identified in each mutant were verified by Sanger sequencing using the Applied Biosystems 3130xl genetic analyzer (Thermo Fisher Scientific, MA, USA).

### Construction of the phylogenetic tree

To construct the OS-MRSA phylogenetic tree, kSNP3^69^, available at https://sourceforge.net/projects/ksnp/, was first used to identify single nucleotide polymorphisms (SNPs) in the whole-genome sequencing data of OS-MRSA strains. The k-mer size was set to an optimum length of 13 as estimated by Kchooser for extracting SNPs from the sequence data. A maximum parsimony tree was then constructed using the majority of the SNPs present in at least 75% of the genomes. The generated phylogenetic tree was visualized using FigTree ver.1.4.3 (tree.bio.ed.ac.uk/software/figtree/).

### Growth curve analysis

The bacterial doubling time was determined as described previously^70^. Briefly, overnight cultures of parent OS-MRSA strains and the laboratory-selected mutants were adjusted to an OD_600_ of 0.2 in MHB. Then, aliquots of 100 µL were inoculated into 10 mL of MHB (final concentration of 1 × 10^5^ CFU/mL), and the cultures were grown at 37 °C with agitation at 25 rpm in an automatic temperature gradient rocking incubator (model TVS126MB; Advantec, Tokyo, Japan). The cell densities at OD_600_ were measured every 5 min for 12 h, and the bacterial growth curve was generated by plotting the measured ODs against time. The doubling time was determined by fitting the growth curve to an exponential equation. Bacterial growth was measured from at least three independent experiments.

### Determination of intracellular ATP levels

The parent OS-MRSA strains and the laboratory-selected mutants were cultured overnight in MHB at 37 °C with agitation at 150 rpm. The overnight cultures were adjusted to an OD_600_ of 0.2 in MHB, and 100 µL of the OD-adjusted culture were inoculated into 10 mL of MHB. The cultures were grown at 37 °C with agitation at 25 rpm in an automatic temperature gradient rocking incubator. One mL of each mid-exponential phase culture (OD_600_ = 0.5) was then transferred to a clean 1.5-mL tube and immediately centrifuged at 15,000 rpm for 1 min at 4 °C to pellet cells. After centrifugation, the cell pellet was stored immediately at − 80 °C until analysis. To determine intracellular ATP levels, a BacTiter-Glo Microbial Cell Viability Assay kit (Promega, WI, USA) was used according to the manufacturer’s instructions. Briefly, the cell pellet was resuspended in 1 mL of MHB, and 25 µL of the cell suspension were mixed with an equal volume of BacTiter-Glo Reagent in a 384-well opaque plate (Iwaki, Tokyo, Japan) and incubated at room temperature for 5 min. The luminescence was then read on an EnVision 2104 Multilabel Reader (Perkin Elmer, Waltham, MA, USA). The ATP concentration was determined with reference to an ATP standard curve prepared from ATP disodium salt hydrate (A2383, Merck KGaA, Darmstadt, Germany). ATP disodium salt was dissolved in distilled water, yielding 1 µM ATP standard solutions. Serial tenfold dilutions of the ATP standard solution were created using MHB to prepare diluted standards that were then used to generate the standard curve. The intracellular ATP concentration of each sample was presented as the mean of three independent experiments performed using three biological replicates.

### RNA extraction

Overnight cultures of the parent OS-MRSA strains and the laboratory-selected mutants were adjusted to an OD_600_ of 0.4. The OD-adjusted cultures were then diluted 1:100 in 1 or 10 mL of MHB for qRT-PCR and RNA-seq analysis, respectively. Each culture was grown to the early log-phase (OD_600_ = 0.3) before treatment with a final concentration of 0.1 μg/mL oxacillin or equal volume of distilled water (control) for 1 h (qRT-PCR) or until OD_600_ = 0.6 (RNA-seq). After oxacillin treatment, the bacterial cells were harvested by centrifugation at 15,000 rpm for 1 min at 4 °C (qRT-PCR) or at 8000 rpm for 5 min at 4 °C (RNA-seq). Pelleted cells were resuspended in 600 μL (qRT-PCR) or 6 mL (RNA-seq) of TE buffer (10 mM Tris–HCl and 10 mM EDTA, pH 8.0) and lysed with 25 (qRT-PCR) or 30 µg (RNA-seq) of lysostaphin (Merck KGaA) by incubating the mixtures at 37 °C for less than 5 min. Total RNA was then extracted using acidic-phenol saturated with 20 mM sodium acetate (pH 4.8) and chloroform and enriched via ethanol precipitation. Contaminating DNA was removed from the total RNA preparations by incubating the solutions with 2 (qRT-PCR) or 20 units (RNA-seq) of RNase-free DNase I (F. Hoffmann-La Roche Ltd, Basel, Switzerland) at 37 °C for 30 min. Total RNA was finally purified using acidic-phenol/chloroform and eluted in RNase-free water.

### Determination of *mecA* levels by qRT-PCR

The extracted total RNA (100 ng per sample) was reverse-transcribed into complementary DNA (cDNA) using a PrimeScript 1st Strand cDNA Synthesis Kit (Takara Bio). qRT-PCR was performed using TB Green *Premix Ex Taq* (Tli RNaseH Plus, Takara Bio) on the Mx3005P Real-Time PCR instrument (Stratagene, La Jolla, CA, USA). A primer set (*mecA*-F-qRT-PCR and *mecA*-R-qRT-PCR, Table S5) was used to amplify the 162-bp *mecA* sequence, whereas the 163-bp housekeeping gene *rho* was amplified using designated primers (*rho*-F-qRT-PCR and *rho*-R-qRT-PCR, Table S5) and used as the reference gene for normalization during gene expression analysis. The thermal cycling conditions included initial denaturation at 95 °C for 30 s followed by 40 cycles of 95 °C for 5 s and 60 °C for 30 s.

### RNA-seq analysis

To perform RNA-seq analysis, ribosomal RNAs (rRNAs) in total RNA preparations of the JMUB217 strain and its mutant derivatives were first depleted using a Ribo-Zero rRNA Removal Kit (Bacteria) from Illumina. Double-stranded cDNA was then synthesized using a PrimeScript Double Strand cDNA Synthesis Kit (Takara Bio). The generated cDNA served as the template for constructing the paired-end library using a Nextera XT library prep kit, and the library was subsequently sequenced using the Illumina MiSeq platform and the MiSeq reagent kit version 3. RNA-seq analysis was performed using CLC Genomics Workbench version 9, and the RNA-seq reads were aligned to the reference genomes of the parent strain JMUB217. Gene expression was normalized by calculating the reads per kilobase per million mapped reads, and differentially expressed genes were identified using Baggerly’s test (β-binomial test) with false discovery rate correction. Genes with adjusted *p* < 0.01 were considered to be significantly differentially expressed.

### Construction of *mecA-* and *blaI*-knockout mutants

To construct *mecA* and *blaI*-knockout mutants of the JMUB217 strain, the pKFT markerless gene deletion system was used as previously described^71^. Briefly, to delete *mecA*, 1-kb upstream and downstream flanking sequences of the target gene were amplified by PCR using the primer sets SacI-mecAKO-UP-2/mecA_fPCR_UP and PstI-mecAKO-UP/mecA_fPCR_DN (Table S5), respectively, with KOD FX Neo (Toyobo). Then, second-round PCR was performed using the first-round PCR products as templates with the primer set SacI-mecAKO-UP-2/PstI-mecAKO-UP. The second-round PCR products and pKFT were digested with the restriction enzymes *Pst*I and *Sac*I (Takara Bio) and ligated using Ligation high ver. 2 (Toyobo), generating the plasmid pmecAKO. pmecAKO was transformed into *E. coli* DH5α, and the transformed cells were plated on LB agar with 100 µg/mL ampicillin. Regarding the generation of *blaI*-knockout mutants, DNA fragments containing *blaI*-1 (locus tag: JMUB217_1395) or *blaI*-2 (locus tag: JMUB217_2048) were first amplified with the primer sets BlaI-1-1/BlaI-1,2-2 and BlaI-2-1/BlaI-1,2-2 (Table S5), respectively. The PCR fragments and pKFT were then digested using the restriction enzymes *Bam*HI and *Pst*I (Takara Bio) and ligated using Ligation high ver. 2. The ligated DNA fragments were independently transformed into *E. coli* DH5α, and the transformed cells were plated on LB agar with 100 µg/mL ampicillin. The plasmids were extracted, and second-round PCR was conducted using the primer set BlaI-1,2-3/BlaI-1-4 for *blaI*-1 knockout and BlaI-1,2-3/BlaI-2-4 for *blaI*-2 knockout. The self-ligated PCR fragments (pblaI-1KO and pblaI-2KO) were again individually transformed into *E. coli* DH5α, and transformed cells were plated on LB agar with 100 µg/mL ampicillin. Afterwards, all three plasmids (pmecAKO, pblaI-1KO, and pblaI-2KO) were extracted from the *E. coli* DH5α transformants and transformed into *E. coli* BL21. The plasmids extracted from *E. coli* BL21 were subsequently electroporated into *S. aureus* JMUB217 and mutants with reduced oxacillin susceptibility as previously described^72^, and the cells were cultured on TSA with 5 µg/mL tetracycline at 30 °C. An isolated colony was then grown overnight in 4 mL of TSB containing 5 µg/mL tetracycline at 30 °C. Single crossover was performed by growing the overnight culture on TSA with 5 µg/mL tetracycline at 43 °C. Then, double crossover was performed by incubating the single crossover mutant on TSA at 30 °C. The double crossover event was confirmed by PCR and Sanger sequencing.

### Complementation of *mecA*

To generate a *mecA*-complemented mutant, a DNA fragment containing wild-type *mecA* from strain JMUB217 was amplified using the primers *Sma*I-mecAcomp-F-pKAT and *Sal*I-mecAcomp-R-pKAT (Table S5). The PCR fragment and pKAT were digested with *Sma*I and *Sal*I (Takara Bio) and ligated using Ligation high ver. 2. The ligated DNA fragment was transformed into *E. coli* DH5α, and the transformed cells were plated on LB agar with 10 µg/mL chloramphenicol. Finally, the complementation plasmid was extracted and electroporated into the JMUB217 strain^72^.

### Statistical analysis

All statistical analyses were performed using Prism 8 (GraphPad Software, San Diego, CA, USA). Statistical comparison was carried out using the Student’s t-test whereas the correlations between variables were calculated using the non-parametric Spearman’s correlation coefficient (*r*_*s*_). Statistical significance was denoted with a *P *value of < 0.05.

## Supplementary information

Supplementary Table S1.

Supplementary Table S2.

Supplementary Table S3.

Supplementary Table S4.

Supplementary Table S5.

## Data Availability

The raw sequence data have been deposited in DNA Data Bank of Japan (DDBJ) under accession number DRA009699 and DRA009727.
